# 15-deoxy-Δ^12,14^-prostaglandin J_2_ in neurodegenerative diseases and cancers

**DOI:** 10.18632/oncotarget.14701

**Published:** 2017-01-17

**Authors:** Tatsurou Yagami, Yasuhiro Yamamoto, Hiromi Koma

**Affiliations:** Faculty of Pharmaceutical Sciences, Himeji Dokkyo University, Japan

**Keywords:** 15-deoxy-Δ^12,14^–prostaglandin J_2_, phosphoinositide 3-kinase, neurodegeneration, cancer, memory retrieval, Neuroscience

**Figure 1:**
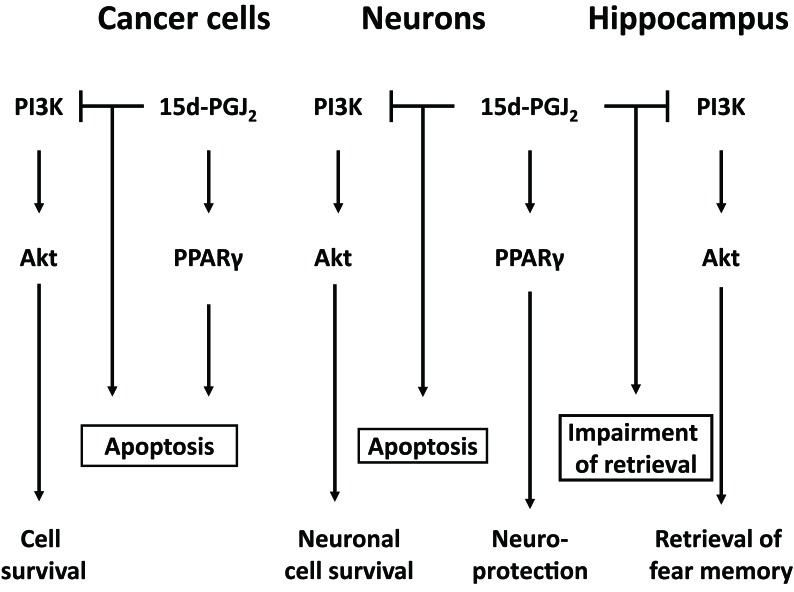
Down-regulation of PI3K pathways is involved in anticancer activities and neurotoxicities of 15d-PGJ_2_. 15d-PGJ_2_ induces neuronal apoptosis via inactivation of PI3K, which is required for cell survival and hippocampal memory retrieval

Neurodegenerative diseases such as Alzheimer’s disease (AD) and Parkinson’s disease (PD) appear to have no connection with cancers. In the view of cell death, however, common ground can be found between neuronal and non-neuronal diseases [1]. AD and PD are ascribed to the cell death of neurons, which should be alive under healthy conditions. In contrast, cancers are attributed to the proliferation of abnormal cells, which should be dead appropriately. The uncontrollability of cell death contributes to the pathogenesis of these diseases. Are there endogenous ligands related to these diseases? An endogenous anticancer 15-deoxy-Δ^12,14^-prostaglandin J_2_ (15d-PGJ_2_) has been the common factor associated with cancers and neurodegenerative diseases (Figure 1). In cancer cells, 15d-PGJ_2_ has been reported to induce apoptosis, which was dependent on or independent of its nuclear receptor, peroxysome-proliferator activated receptor γ (PPARγ) [1, 2].

PD is characterized by the loss of dopaminergic neurons projecting from substantia nigra pars compacta to striatum, and disables motor functions [1]. Subchronic microinfusion of a precursor of 15d-PGJ_2_, PGJ_2_, into the substantia nigra/striatum has been reported to induce PD-like features [3]. Unilateral lesions of the dopaminergic nigrostriatal system caused circling behavior, which was ascribed to the functional imbalance between the dopaminergic nigrostriatal pathways on the two sides of the brain. We confirmed that the unilateral injection of 15d-PGJ_2_ into striatum induced circling in the ipsilateral direction (data not shown). Thus, 15d-PGJ_2_ could act as a neurodegenerative mediator of PD.

Among various PGs, a precursor of 15d-PGJ_2_, PGD_2_, is most abundant in the central nervous system, and increased in cerebral cortex of AD patients [4]. AD is characterized pathologically by neurofibrillary tangles and senile plaques, which are followed by neuronal loss and cortical atrophy [1]. In senile plaques of various brain regions such as cerebral cortex and hippocampus, aggregated deposits of amyloid β protein (Aβ) are considered to play a causative role in neurodegeneration and development of AD. Aβ induced neuronal cell death via apoptosis accompanied with PGD_2_ production [5]. Aβ- induced apoptosis and PGD_2_ were suppressed by inhibitors of cyclooxygenase-2, which is the inducible enzyme hydrolyzing arachidonic acid to PGG_2_ and _2_. PGD_2_ is non-enzymatically metabolized to 15d-PGJ_2_, which possesses opposite functions as a neuroprotectant at low concentrations and a neurotoxicant at high concentrations [1]. In the central nervous system, PPARγ mediates the neuroprotective effect of 15d-PGJ_2_ (Figure 1), whereas neither PPARγ nor its membrane receptor, chemoattractant receptor-homologous molecule expressed on T-helper type 2 cells is not involved in the neurotoxicity of 15d-PGJ_2_ [1].

15d-PGJ_2_ inhibits growth factor-induced cell proliferation of primary astrocytes, neuroblastoma and carcinomas via down-regulating phosphoinositide 3-kinase (PI3K)-Akt pathway [6]. As shown in Figure 1, this pathway has been also required for neuron to survive regardless serum throughout maturation [7]. 15d-PGJ_2_ disrupted neuronal cell bodies, shortened neurites thinly, damaged plasma membranes and activated caspase-3 similarly to the PI3K inhibitor. The PI3K signaling is essential for enduring forms of synaptic plasticity underlying learning and memory [8]. In the hippocampus, bilateral injection of 15d-PGJ_2_ impaired contextual memory retrieval requiring the PI3K signaling [7]. A PI3K activator suppressed the 15d-PGJ_2_-induced cell death and -impaired memory retrieval. In neurons as well as cancer cells, 15d-PGJ_2_ exhibited cytotoxicity via suppressing the PI3K-Akt pathway. Thus, 15d-PGJ_2_ is identified not only as the endogenous anticancer agent in the peripheral tissues, but also recognized as one of the neurodegenerative mediators in the central nervous system.
